# Rediscovery and redescription of *Dilobitarsus pendleburyi* (Coleoptera, Elateridae, Agrypninae) from Southeast Asia

**DOI:** 10.3897/zookeys.593.7995

**Published:** 2016-05-26

**Authors:** Kôichi Arimoto

**Affiliations:** 1 Entomological Laboratory, Graduate School of Bioresource and Bioenvironmental Sciences, Kyushu University, Fukuoka, 812–8581 Japan

**Keywords:** Agrypnini, new distributional records, Oriental region, Sumatra, taxonomy, the Malay Peninsula

## Abstract

*Dilobitarsus pendleburyi* Fleutiaux, 1934 is recorded for the first time after its original description and is redescribed. This represents the first record from the Malay Peninsula, Malaysia and Sumatra, Indonesia. The systematic position of this species is discussed.

## Introduction

The genus *Dilobitarsus* Latreille, 1834 is represented by 32 species belonging to Agrypnini. Among them, 31 species are distributed in the New World, and only one species *Dilobitarsus pendleburyi* Fleutiaux, 1934 is found in the Oriental region (Casari 2013). Almost all species are known only from their original descriptions, and their genitalia and mouth-parts are not described (e.g., Candèze 1857; Schwarz 1902; Fleutiaux 1907). Hayek (1973) reviewed this genus by examining type and non-type specimens, but did not find some type specimens (perhaps lost), or include species descriptions or illustrations. Casari (2013) described four new species in detail including descriptions of the genitalia and mouth parts. She provided a key to the New World species. This research improved our understanding of the morphology of *Dilobitarsus*. Fleutiaux (1934) described *Dilobitarsus pendleburyi* from a male specimen from Borneo, Malaysia. No subsequent records or information have been published. I examined the type specimen of this species and additional specimens including a male from the Malay Peninsula, Malaysia and a female from Sumatra, Indonesia. This paper redescribes this species, and presents new distributional records from the Malay Peninsula and Sumatra. The systematic position of this species is discussed.

## Materials and methods

The type specimen is deposited in the Natural History Museum, London (BMNH). Non-type specimens examined are in the personal collection of Kôichi Arimoto and Hisayuki Arimoto (CAR; Osaka, Japan).

Photographs of specimens were taken using a single-lens reflex camera (Canon EOS 7D) with a macro lens (Canon macro photo lens MP-E 65-mm) and combined using image processing software (CombineZM, Alan Hadley).

The morphology of specimens was observed under a stereo microscope (Olympus-SZX9). Measurements are in millimeters and were made with a micro ruler (MR-2, minimum scale value: 0.05 mm, Kenis Limited, Ôsaka, Japan) to obtain the following properties: body length from apex of the head to apices of the elytra (BL), body width (BW), pronotum length including posterior angles (PL), length of the midline of the pronotum (PML), pronotum width including posterior angles (PW), elytra length (EL), and elytra width (EW). Non-type specimens were used for dissection. The mouth-parts, pregenital segments and genitalia were soaked in 10% KOH solution (room temperature, male: 2 hours, female: 30 hours). The parts were dehydrated in 99.5% ethanol (5 min) and then mounted in euparal on a microscope slide, except for mounting of the bursa copulatrix in glycerin. A transmission microscope (Nikon Y-IDT) attached to a drawing device was used for observations of the dissected parts and creation of line drawings. Morphological terminology follows Calder (1996), and Casari (2013) in part.

Maps were made using free software (DIVA-GIS 7.5.0.). The digital images of map, photographs and drawings were edited with image editing software (Adobe Photoshop 7.0).

## Taxonomy

### Dilobitarsus pendleburyi

Taxon classificationAnimaliaColeopteraElateridae

Fleutiaux, 1934

Figures 1 , 2–6 , 7–11 , 12–16 , 17–22 , 23–29 , 30–38

Dilobitarsus pendleburyi Fleutiaux, 1934: 178 (original description; type locality: Near Sandakan, Bettotan, Sabah, Northern Borneo, Malaysia); Hayek 1973: 99 (generic review; examination of the holotype).

#### Type material.

**Holotype**: Male, 13 VIII 1927, Near Sandakan, Bettotan, Sabah, Northern Borneo, Malaysia. (BMNH).

#### Non type materials.

1 male, Fraser’s Hill, Pahang, Malaysia, 9 V 2010, K. Matsuda leg. (CAR); 1 female, Harau Valley, near Payakumbuh, West Sumatra, Indonesia, 15 VIII 1992, A. Sarimudanas leg. (CAR).

#### Distribution

**Figure 1.**
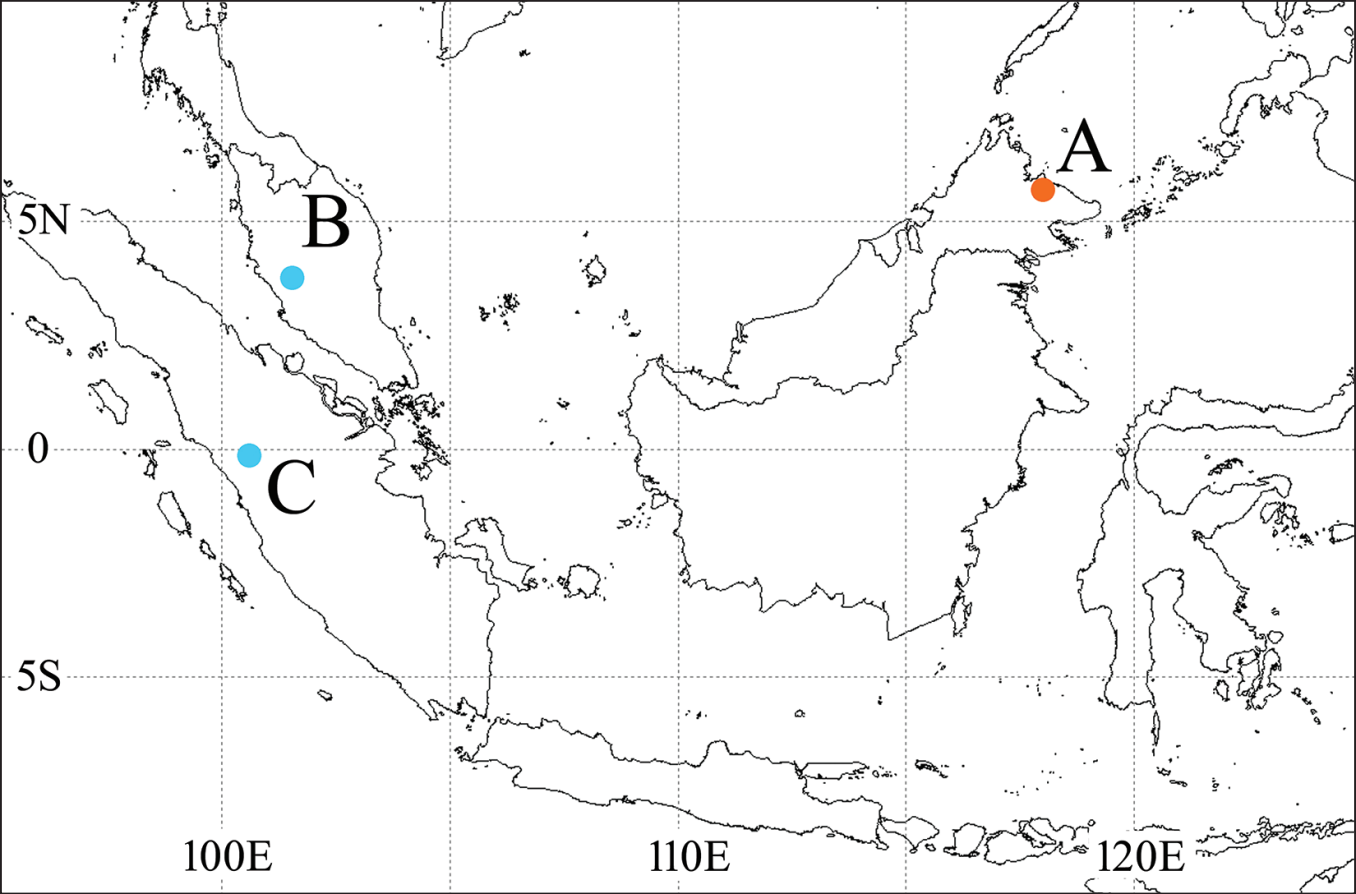
Collection localities of *Dilobitarsus pendleburyi* Fleutiaux, 1934. **A** Bettotan, Sabah, Northern Borneo, Malaysia (type locality) **B** Fraser’s Hill, Pahang, Malaysia; C: Harau Valley, West Sumatra, Indonesia.

(Fig. 1). Oriental Region: Malaysia (Borneo, the Malay Peninsula), Indonesia (Sumatra). New records from the Malay Peninsula and Sumatra.

#### Measurements.

Male [holotype]; BL: 11.75 [11.51], BW: 3.14 [3.03], PL: 3.61 [3.58], PML: 3.04 [3.20], PW: 2.89 [2.91], PL/PW: 1.25 [1.23], EL: 7.76 [7.53], EW: 3.14 [3.03], EL/EW: 2.47 [2.49]. Female; BL: 15.21, BW: 3.92, PL: 4.70, PML: 4.08; PW: 3.77, PL/PW: 1.25, EL: 9.65, EW: 3.92; EL/EW: 2.46.

#### Diagnosis.

**Figures 2–6.**
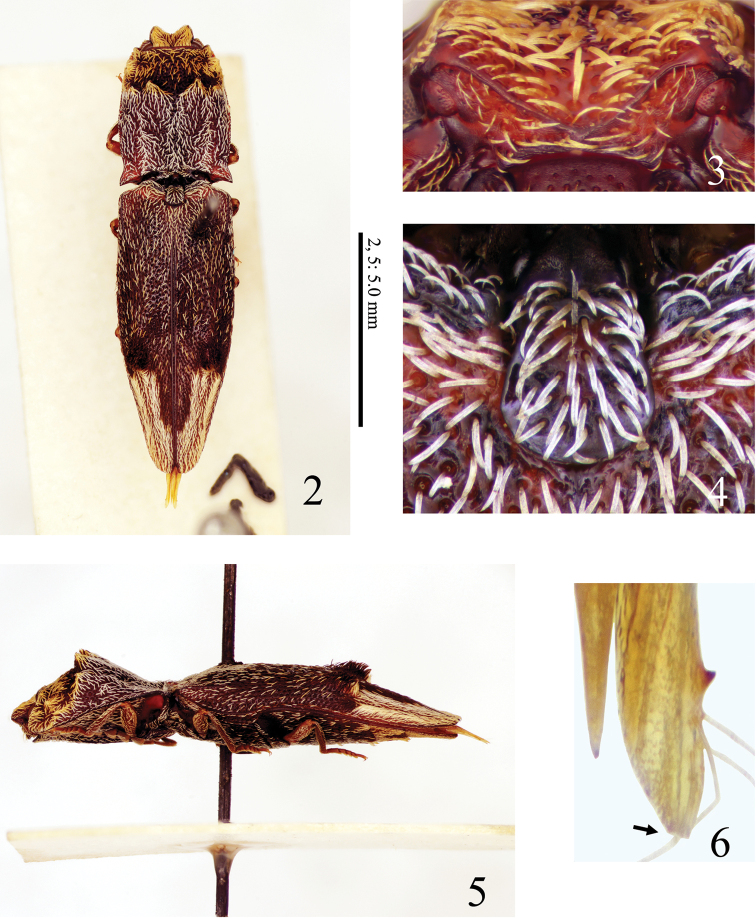
*Dilobitarsus pendleburyi* Fleutiaux, 1934, holotype, male. **2** Habitus, dorsal view **3** head, anterior view **4** scutellum **5** habitus, lateral view **6** paramere apex of aedeagus.

**Figures 7–11.**
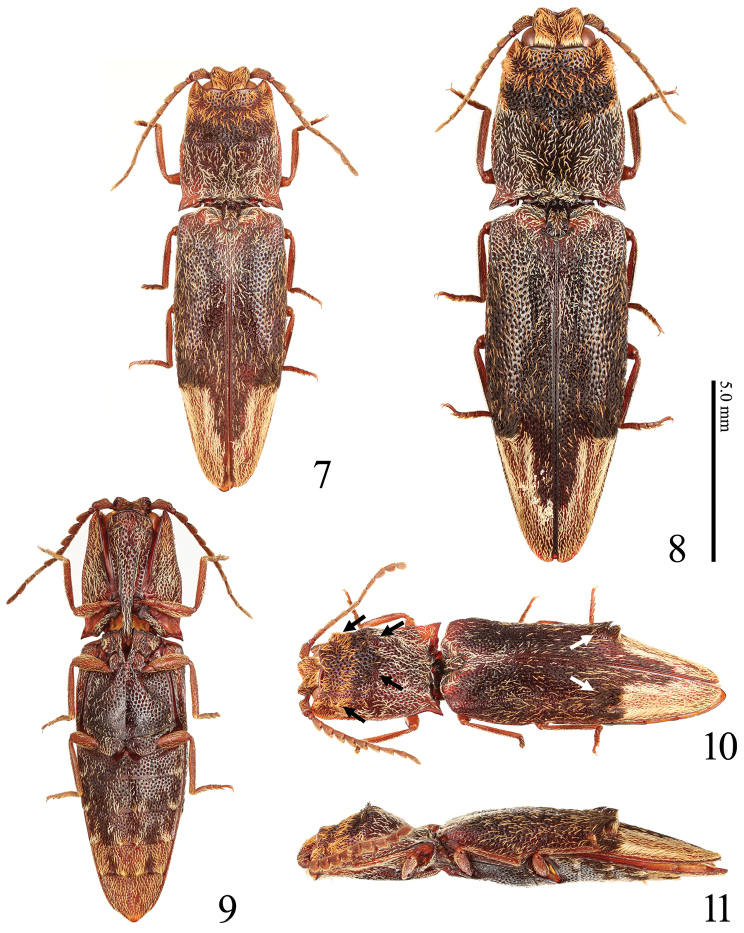
Habitus of *Dilobitarsus pendleburyi* Fleutiaux, 1934. **7** Male, dorsal view **8** female, dorsal view **9** male, ventral view **10** male, dorsolateral view **11** male, lateral view.

**Figures 12–16.**
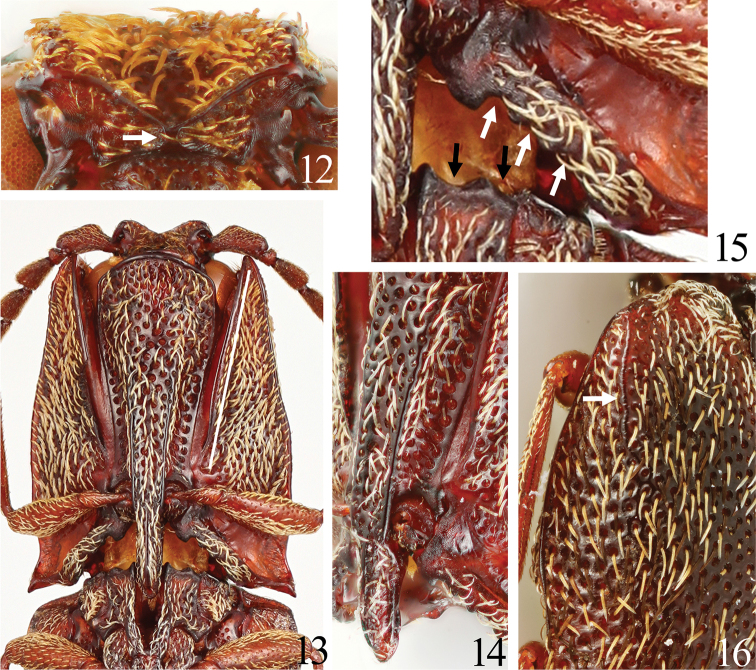
*Dilobitarsus pendleburyi* Fleutiaux, 1934, male. **12** Head, anterior view of clypeus **13** prothorax, ventral view **14** prothorax, ventrolateral view **15** posterior margin of hypomeron and mesosternam; 16: humerus of elytra.

Setae narrow and scale-like in black, white and orange (Figs 2, 7, 8); head with frontal carina V-shaped (Figs 3, 12); nasal plate high laterally and divided medially by a short vertical carina (Figs 3, 12, arrow); pronotum with four tubercles (Fig. 10, black arrows); hypomeron concave longitudinally along pronotosternal suture (Fig. 13, white line); posterior margin of hypomeron with three notches at inside (Fig. 15, white arrows); metasternum depressed for reception of mid tarsi; elytra with two tubercles (Fig. 10, white arrows); parameres of aedeagus not constricted basal to lateral subapical barb (Figs 27–29); apex long (Figs 27, 28); sclerotized plate in bursa copulatrix U-shaped, and with long teeth (Figs 36–38).

**Figures 17–22.**
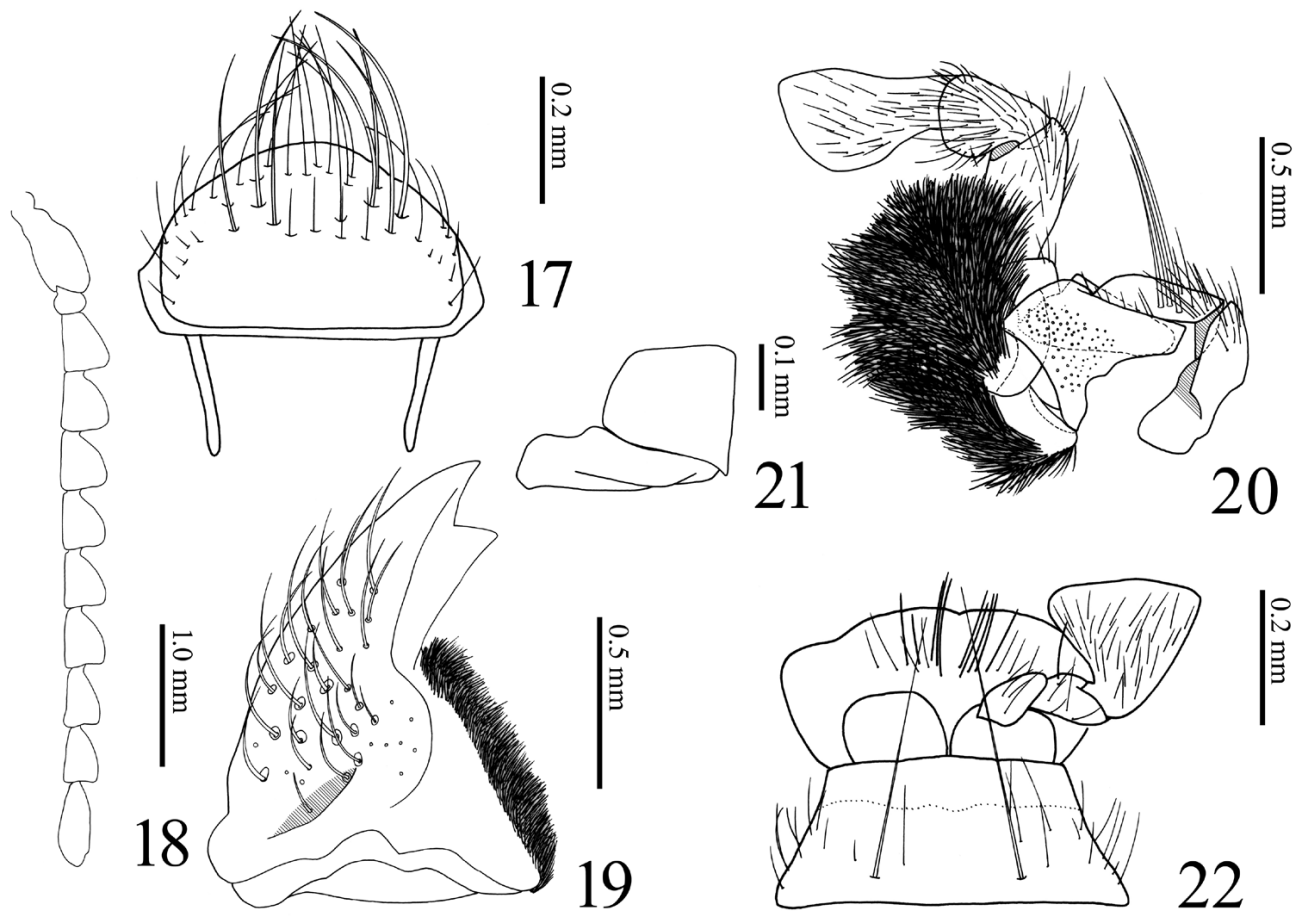
*Dilobitarsus pendleburyi* Fleutiaux, 1934, male. **17** Labrum **18** antenna **19** mandible, dorsal view **20** maxilla **21** stipes, ventral view **22** labium.

**Figures 23–29.**
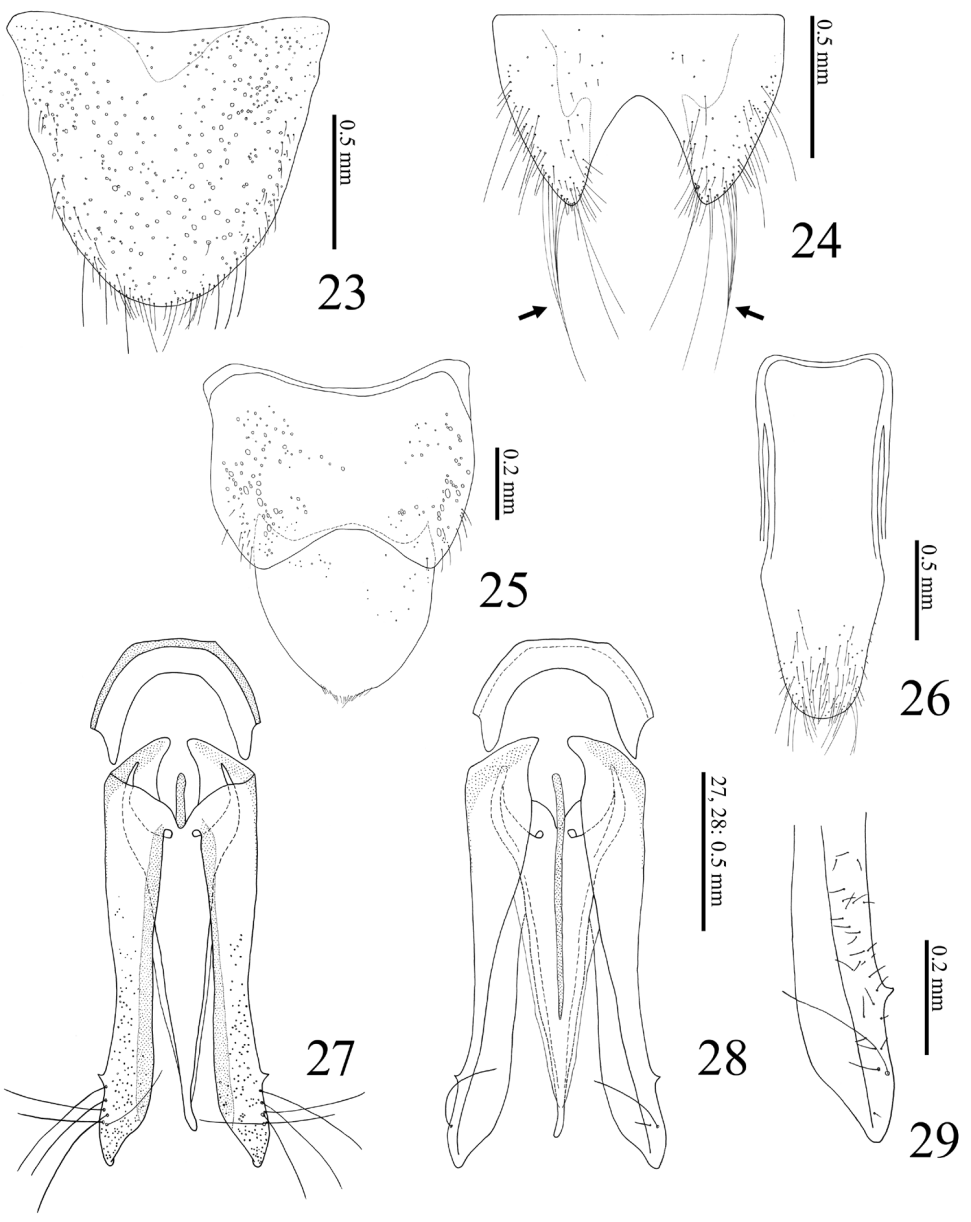
*Dilobitarsus pendleburyi* Fleutiaux, 1934, male. **23** Terigite VIII **24** sternite VIII **25** tergites IX–X **26** sternite IX **27** aedeagus, dorsal view **28** ditto, ventral view **29** apical part of paramere, ventral view.

**Figures 30–38.**
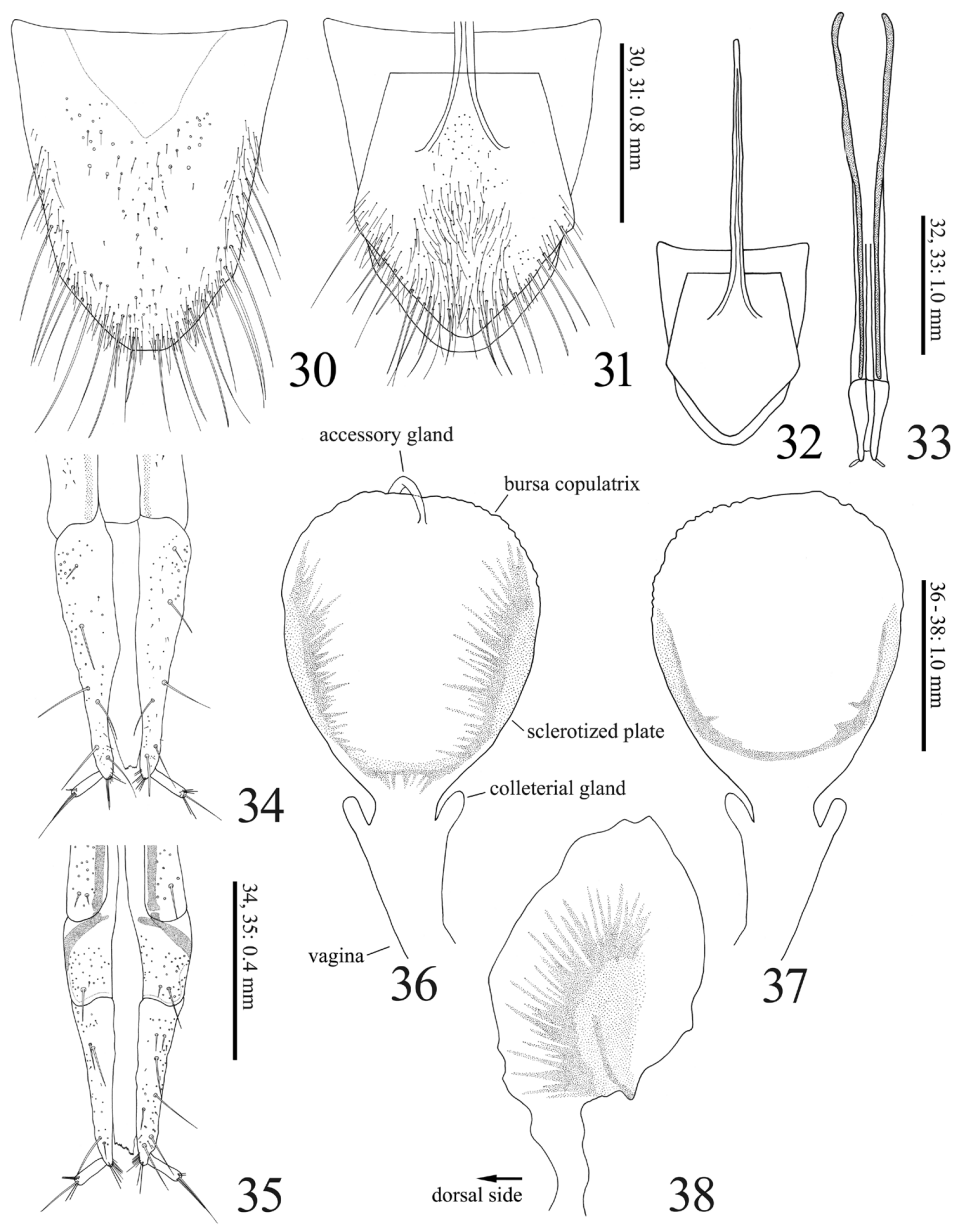
*Dilobitarsus pendleburyi* Fleutiaux, 1934, female. **30** Tergite VIII **31, 32** sternite VIII and tergite VIII (ventral) **33** ovipositor, dorsal view **34** apex of ovipositor, dorsal view **35** ditto, ventral view **36** bursa copulatrix, dorsal view **37** ditto, ventral view **38** ditto, lateral view.

#### Redescription.

Adult. Body (Figs 2, 7, 8) elongate, convex; surface smooth, shining, with large punctures; black-brown to red-brown, but mouth-parts yellow-brown with mandible black-brown. Setae. Body covered with narrow scale-like setae; bases of tarsal claws each with a thick seta. Head and pronotal anterior part with orange setae; pronotum with black setae at the center, with white setae posteriorly; elytra with intermixed white and black setae, together with white U-shaped setal apical band; setae at pronotal and elytral tubercles denser and erect; ventral surface with white setae, but abdominal ventrites partially with dense orange setae (Fig. 9).

Frons (Figs 3, 12) depressed mesally; frontal carina V-shaped in anterior view; nasal plate high laterally (higher in non-type), divided medially by a short vertical carina (longer in non-type, Fig. 12, arrow). Labrum (Fig. 17) semicircular, with long setae apically. Antennae (Fig. 18); ***male***: extended beyond pronotal posterior apices by length of antennomere 11; ***female***: not reaching pronotal posterior apices by distance equal to length of antennomere 11; relative antennomere lengths: II< III-X< XI< I; antennomere I cylindrical; antennomeres III-X serrate; antennomere XI oblong ovate. Mandible (Fig. 19) bidentate; penicillum developed, formed by dense short setae; dorsal sinuous carina developed; concave ahead of posterior condyle. Maxilla (Fig. 20); basistipes subrectangular (Fig. 21), with three long setae; mediostipes with sinuous posterior margin, with a median longitudinal carina (Fig. 21); galea and lacinia brush-like; palpus short, pilose. Labium (Fig. 22); mentum trapezoidal, translucent anteriorly, with two long setae; prementum with rounded anterior margin, with a median anterior notch shallow, with a transverse row of setae; apical palpomere hatchet-shaped, short.

Prothorax longer than wide; anterior angles acute; lateral carina complete; sides rounded posteriorly, constricted anterior to hind angles. Pronotum; disk with four tubercles elevated strongly (Fig. 5) (to weakly, Fig. 10, black arrows); punctures at tubercles small and dense; posterior angles short, without carina; base elevated medially. Prosternum (Fig. 13) convex medially, with carinae extending anterad of coxal cavities (Fig. 14); anterior edge carinate, with anterior lobe prominent, reaching to level of apices of anterior angles of prothorax; prosternal spine weakly inclined dorsally behind procoxae, flattened laterally, ventral surface carinate medially (Fig. 14), with ventral and dorsal apices rounded. Hypomeron concave longitudinally along pronotosternal suture (Fig. 13, white line); posterior edge carinate behind depression for reception of proleg, with three notches mesally (Fig. 15, white arrows); punctures smaller than on prosternum. Pronotosternal sutures deeply grooved and receive antennae, opened anteriorly; antennal groove becoming shallower posteriad (Fig. 13). Scutellum tongue-shaped (Fig. 4), longer than wide, flat; narrowed at anterior third; apex round; punctures small. Mesosternum with two concavities on anterior margin (Fig. 15, black arrows); concave anteriorly in lateral view (Figs 5, 11). Mesocoxal cavity closed to mesepimeron. Suture between mesosternum and metasternum present. Metasternum (Fig. 9) sulcate medially and behind mesocoxae; punctures smaller posteriad. Elytra broadly convex, without striae; with two tubercles elevated strongly (Fig. 5) (to weakly, Fig. 10, white arrows); apices rounded; punctures smaller laterad; with a longitudinal carina at each humerus (Fig. 16, white arrow). Tibiae without spurs; relative tarsomere lengths: IV< III< II< V< I; ventral lobes not developed at tarsomeres II and III, longer at tarsomere IV; tarsal claws simple.

Abdomen. ***Male*.** Tergite VIII (Fig. 23) wider than long; translucent in medina basal area; posterior margin setose. Sternite VIII (Fig. 24) emarginate, wide; posterior notch large; translucent except with yellow band on each side; some long setae bunched together (Fig. 24, arrows). Tergite IX (Fig. 25) with posterior notch shallow; with some short setae on posterior angles. Tergite X (Fig. 25) semicircular; apical margin with fine setae. Sternite IX (Fig. 26) long; sides constricted medially. ***Female*.** Tergite VIII (Fig. 30) longer than wide, translucent in median basal area; basal margin membranous and indefinite; lateroapical margin fringed with long setae. Sternite VIII (Fig. 31) shield-shaped, longer than wide; basal margin membranous; apical margin fringed with long setae; spiculum ventrale 1.7 X length of sternite VIII (Fig. 32).

Genitalia. ***Male*.** Aedeagus (Figs 27–29) elongate. Median lobe not exceeding apices of parameres; apex slender. Parameres separated ventrally, not constricted anterior to lateral subapical barb; apex beyond lateral subapical barb 0.2 X length of paramere, with four (to six) long setae dorsally (Figs 6, 27) and one long setae and some short setae ventrally (Fig. 28); apex truncate transversal (Fig. 6: arrow) (or rounded, Fig. 29). ***Female*.** Ovipositor (Fig. 33) slender. Each coxite two-segmented ventrally (Fig. 35), with four long and two short setae at dorsal side (Fig. 34), with some thick setae (14 setae recognized in specimen examined) at ventral side (Fig. 35); apex with dense setae. Stylus setose. Colleterial gland not developed (Figs 36, 37). Bursa copulatrix (Figs 36, 37) globular, large; anteriormost part with a short accessory gland; sclerotized plate U-shaped, large and with long teeth (Figs 36, 38).

#### Larvae and pupae.

Unknown.

#### Remarks.

This species is easily identified by its three-coloured setal pattern and tubercles of the pronotum and elytra.

#### Bionomics.

Nothing is known about the life history.

## Discussion

*Dilobitarsus* Latreille, 1834 was placed in tribe Agrypnini (sensu Stibick 1979) of the subfamily Agrypninae because its pronotosternal sutures are deeply grooved and recieve the antennae (Fig. 13), the prosternum, mesosternum and mesepisternum are simple, mesepimeron not reduced (Fig. 13), and setae on base of the claws. In Southeastern Asia the tribe contains nine genera: *Agrypnus* Eschscholtz, 1829; *Adelocera* Latreille, 1829; *Dilobitarsus*; *Lacon* Castelnau, 1836; *Meristhus* Candèze, 1857; *Danosoma* Thomson, 1859; *Octocryptus* Candèze, 1892; *Rismethus* Fleutiaux, 1947; and *Lanelater* Arnett, 1952 (Hayek 1973). *Dilobitarsus* is separated these by the combination of the following characteristics (Arnett 1952; Arnett et al. 1969; Hayek 1973): large body length (over 10 mm), body covered with scale-like setae, antennomere III longer than II (Fig. 18), hypomeron without longitudinal grooves near the lateral margins (Fig. 13), prothorax not constricted behind the anterior angles (Fig. 13), scutellum without longitudinal carina (Fig. 4), middle coxal cavity reaching mesosternum and mesepimeron (Fig. 13), tibial spurs absent and tarsal segments with ventral lobes. Presence of ventral tarsal lobes has been treated as important for generic diagnosis. *Dilobitarsus* and its species have been described from several continents based on this state (e.g., Candèze 1857). Schwarz (1902) established *Elasmosomus* and transferred some African *Dilobitarsus* species there. Fleutiaux (1934) did not recognize *Elasmosomus* and described *Dilobitarsus pendleburyi* from Borneo in Malaysia as the only Oriental species of *Dilobitarsus*. Hayek (1973) transferred two African *Dilobitarsus* species to *Elasmosomus*. Consequently, all *Dilobitarsus* species described from Africa have been assigned to *Elasmosomus*, and only the single Oriental species remains in *Dilobitarsus*.

*Dilobitarsus* appear to be closely related to the seven genera *Lacon*, *Hemicleus* Candèze, 1857, *Danosoma*, *Eidolus* Candèze, 1857, *Acrocryptus* Candèze, 1874, *Elasmosomus* Schwarz, 1902 and *Candanius* Hayek, 1973 because all share the all above character states except for ventral tarsal lobe, and they belong to the informal *Dilobitarsus*-genus group (here proposed). The genera of this group are characterized especially with antennomere III larger than II (Fig. 18) in Agrypninae. Although *Dilobitarsus*, *HemicleusAcrocryptus* and *Elasmosomus* share ventral tarsal lobes, since this characteristic has been observed in many distant lineages of Elateridae, there is the possibility that it is homoplasy. Additionally, the degree of development of the ventral tarsal lobes varies between species. Future studies of the phylogenetic relationships in Agrypnini and Agrypninae are needed in order to determine whether the presence of ventral tarsal lobes represents homoplasy and to test the monophyly of the *Dilobitarsus*-genus group.

*Dilobitarsus pendleburyi* is characterized especially with V-shaped frontal margin and laterally high nasal plate (Figs 3, 12). It is necessary to review the generic placement of this species. However there is not enough information about head status of the other species. Further reviews of the morphology of the species in this genus group are important to understand precisely the systematic position and apomorphies of *Dilobitarsus pendleburyi*.

## Supplementary Material

XML Treatment for Dilobitarsus pendleburyi
